# Identification and Development of an Age-Related Classification and Signature to Predict Prognosis and Immune Landscape in Osteosarcoma

**DOI:** 10.1155/2022/5040458

**Published:** 2022-10-12

**Authors:** Jinjiong Hong, Xiaofeng Wang, Liang Yu, Jie Li, Haoliang Hu, Weisheng Mao

**Affiliations:** ^1^Department of Hand Surgery, Department of Plastic Reconstructive Surgery, Ningbo No. 6 Hospital, Ningbo 315040, China; ^2^Department of Spine Surgery, Ningbo No. 6 Hospital, Ningbo 315040, China; ^3^Department of Orthopedics, Ningbo Medical Center Lihuili Hospital, Ningbo 315040, China

## Abstract

**Background:**

In childhood and adolescence, the prevailing bone tumor is osteosarcoma associated with frequent recurrence and lung metastasis. This research focused on predicting the survival and immune landscape of osteosarcoma by developing a prognostic signature and establishing aging-related genes (ARGs) subtypes.

**Methods:**

The training group comprised of the transcriptomic and associated clinical data of 84 patients with osteosarcoma accessed at the TARGET database and the validation group consisted of 53 patients from GSE21257. The aging-related subtypes were identified using unsupervised consensus clustering analysis. The ARG signature was developed utilizing multivariate Cox analysis and LASSO regression. The prognostic value was assessed using the univariate and multivariate Cox analyses, Kaplan-Meier plotter, time-dependent ROC curve, and nomogram. The functional enrichment analyses were performed by GSEA, GO, and KEGG analysis, while the ssGSEA, ESTIMATE, and CIBERSORT analyses were conducted to reveal the immune landscape in osteosarcoma.

**Results:**

The two clusters of osteosarcoma patients formed based on 543 ARGs, depicted a considerable difference in the tumor microenvironment, and the overall survival and immune cell infiltration rate varied as well. Among these, the selected 23 ARGs were utilized for the construction of an efficient predictive prognostic signature for the overall survival prediction. The testing in the validation group of osteosarcoma patients confirmed the status of the high-risk score as an independent indicator for poor prognosis, which was already identified as such using the univariate and multivariate Cox analyses. Furthermore, the ARG signature could distinguish different immune-related functions, infiltration status of immune cells, and tumor microenvironment, as well as predict the immunotherapy response of osteosarcoma patients.

**Conclusion:**

The aging-related subtypes were identified and a prognostic signature was developed in this research, which determined different prognoses and allowed for treatment of osteosarcoma patients to be tailored. Additionally, the immunotherapeutic response of individuals with osteosarcoma could also be predicted by the ARG signature.

## 1. Introduction

Osteosarcoma (OS) is the major prevailing bone tumor in childhood and adolescence worldwide that originates from the bone marrow mesenchymal stem cells or osteoclasts [1, 2]. Osteosarcoma occurs mostly in the metaphysis of long bones near an active bone growing region and is generally more prevalent in the femur (42%), the tibia (19%), and the humerus (10%) [3]. The incidence rate of osteosarcoma is relatively low with only 3-4 people being affected per million annually, but the high probability of recurrence and distant metastasis and the absence of identifying symptoms at an early stage together with its highly malignant nature leads to poor prognosis of osteosarcoma patients [4]. Currently, therapeutic management for patients with osteosarcoma mainly depends on surgical resection, chemotherapy, radiation therapy, immunotherapy, and targeted therapy which has caused the 5-year survival rate to increase to 60%-70% in osteosarcoma patients without metastasis, whereas patients with recurrence or metastasis still have a 5-year survival rate less than 30% [5, 6]. The identification of the process behind the occurrence and progression of osteosarcoma is extremely necessary to create effective treatments. The examination of biomarkers to identify effective prognostic markers for osteosarcoma that can enhance the active interventions for the disease and the development of novel therapies may help in increasing the survival rate.

Aging presents the characteristics of gradual deterioration in internal physiological function and is linked to the onset and progression of multiple chronic conditions, including cancers [7]. Cytologically, aging has been linked to the cumulative damage caused by abnormalities such as genomic instability, mitochondrial dysfunction, cellular senescence, which has been related to the development of aging-linked malignancies [8]. Cellular senescence occurs in response to many different triggers, including DNA damage, telomere dysfunction, oncogene activation, and organelle stress, and has been linked to the aging processes [9]. Senescence cells have a highly complex effect on the growth of cancers. The consequent activation of the SASP system results in the secretion of a variety of signaling molecules such as cytokines or chemokines, as well as growth factors, and extracellular matrix proteases which affect tumor growth by either arresting the cell cycle or regulating the immune clearance [10]. The onset of aging-associated malignancies can be delayed by targeting the aging mechanism, which makes the identification of these aging-related markers extremely necessary [11, 12]. The reports from multiple recent studies have demonstrated the involvement of specific genes in modulating cellular senescence, such as *APOE* [13] and *FOXO3* [14]. Peters et al. [15] also performed a population-based large-scale transcriptomic analysis to determine aging-related genes (ARGs). Although osteosarcoma is an age-dependent disease, nevertheless, there is a lack of systemic research on the association between ARGs and the prognosis of osteosarcoma.

In this research, the expression profile of ARGs was utilized to identify two aging-related molecular subtypes in the TARGET database, and the underlying differences between subtypes were systematically revealed. Afterward, the ARGs associated with independent prognosis were filtered out, and an ARGs prognostic signature was constructed to provide a new method for assessing clinical outcomes in patients with osteosarcoma, which was further verified utilizing the Gene Expression Omnibus (GEO) dataset, GSE21257. Moreover, a predictive nomogram utilized for the prediction of accurate survival rates among patients with osteosarcoma was established comprising the ARG signature and clinical features. Finally, the link between the risk model and the immune infiltration landscape was studied to search for new targeted therapies for osteosarcoma.

## 2. Materials and Methods

### 2.1. Data Source

The RNA-seq expression profiles and corresponding clinical and pathological information of 88 osteosarcoma patients were accessed at TARGET datasets (https://ocg.cancer.gov/programs/target, updated January 16, 2022). Afterward, three patients without prognostic information and one patient without clinicopathological information were excluded, and 84 individuals with osteosarcoma were left in the training set. For the validation set, 53 osteosarcoma patients in the GSE21257 were obtained from the Gene Expression Omnibus database (https://www.ncbi.nlm.nih.gov/geo/query/acc.cgi?acc=GSE21257).  Table 1 demonstrates the relevant clinical data of patients with osteosarcoma studied in this research.

### 2.2. Consensus Clustering

A total of 543 aging-related genes were accessed at the Human Aging Genome Resource dataset [16] (HAGR, https://www.genomics.senescence.info/) and the CellAge dataset (https://genomics.senescence.info/cells/) after the elimination of duplicate genes. The different aging-related molecular subtypes were identified according to the aging-related genes by employing the ConsensusClusterPlus package of R. The increase from 2 to 9 in the clustering variable (*k*) was carried out to select the optimum number of subtypes, and the stability of the results was enhanced by replicating the process 1,000 times.

### 2.3. Functional Enrichment Analyses between Aging-Related Subtypes

The differentially expressed genes (DEGs) were identified utilizing the “limma” R package by applying the criteria |log2FC| ≥0.5 and FDR P <0.05 to study the biological function and pathways between aging-related subtypes. These DEGs were analyzed using the Kyoto Encyclopedia of Genes and Genomes (KEGG) pathway analysis and Gene Ontology (GO) enrichment analyses. The annotation and visualization were carried out by the “clusterProfiler,” “http://org.Hs.eg.db,” “ggplot2,” and “enrichment plot” R packages. The different pathways between aging-related subtypes were assessed as described previously utilizing the Gene Set Enrichment Analysis (GSEA) [17].

### 2.4. Evaluation of Immune Characteristics between Aging-Related Subtypes

The tumor microenvironment (TME) scores such as stromal content (StromalScore), tumor purity, and the degree of infiltration of immune cells (ImmuneScore) were measured utilizing the program ESTIMATE [18]. The immune cells infiltration score and the activity level of pathways associated with the immune system were measured using the single-sample Gene Set Enrichment Analysis (ssGSEA) by applying the “gsva” package.

### 2.5. Construction of an Aging-Related Risk Signature for Osteosarcoma

A prognostic predictive risk model was constructed, and the coefficients were identified using the multivariate Cox regression, which was utilized for predicting the risk scores of individuals with osteosarcoma. For the construction of this model, the relevant genes were identified by univariate Cox regression in the TARGET cohort, and the Least Absolute Shrinkage and Selection Operator (LASSO) regression was applied to define the optimum range of aging-related genes utilized in the model. The prognostic aging-relevant genes were identified using the “survival” package and were optimized in the signature by utilizing the “glmnet” package in the aforementioned analyses. The formula mentioned below was utilized to derive the risk score of each individual in the validation and TARGET cohorts:

(1) $$\begin{array}{c} \text{Risk score}=\overset{n}{\underset{i=1}{\sum }}\text{c}\text{oefficient}\times \text{aging}‐\text{related gene expression}. \end{array}$$

The individuals with osteosarcoma were divided based on the median into two groups: the high- and low-risk groups. The prognostic value of the risk model for individuals with osteosarcoma was analyzed using the principal component analysis (PCA), Kaplan-Meier curves, time-dependent receiver operating characteristics (ROC) curves, C-index, decision curve analysis (DCA) [19], univariate and multivariate Cox regression analysis, and survival subgroup analyses. The risk model performance was verified using the validation cohort, which consisted of 53 osteosarcoma patients from the GSE21257. To determine whether our ARG signature had a superior predictive ability [20–23], four previous signatures were selected with which the values of the following parameters were compared such as time-dependent ROC, C-index, and restricted mean survival time (RMST).

### 2.6. Development of Nomograms to Predict the Outcome of Patients with Osteosarcoma

The nomogram was generated for the prediction of the 1-, 3-, and 5-year rates of survival of individuals with osteosarcoma, and its performance was analyzed using various software. The risk score and clinicopathological parameters such as age, gender, and metastasis were utilized for the prediction of the overall survival (OS) rate by using the “rms” package, while the time-dependent ROC and calibration curves were utilized to analyze the nomogram's performance in prognosis prediction.

### 2.7. Evaluation of Immune Characteristics between High-Risk and Low-Risk Group

The variation in the ESTMATEScore, StromalScore, and ImmuneScore content in the TME and the score of pathways associated with the immune system were analyzed between the two risk groups. The ssGSEA was utilized for the comparison of the pathway scores, whereas the CIBERSORT algorithm [24] examined 22 immune cells to determine their infiltration degree. The treatment response of immune checkpoint inhibitors was predicted by examining the level of expression of several important genes associated with immune checkpoints between the two groups.

### 2.8. Statistical Analysis

The R software 4.1.0 was utilized for statistical analysis and visualization of the data involved in this research. The differences between groups were determined by utilizing Wilcoxon signed-rank and chi-square tests. The Pearson correlation analysis analyzed the link between groups. The significant level was selected as *P* values <0.05.

## 3. Results

### 3.1. Consensus Clustering Analysis Based on ARGs

**Figure 1:**
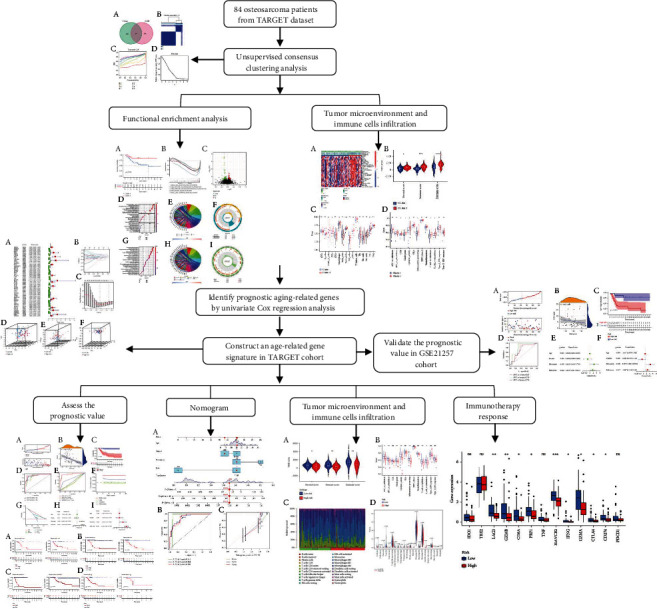
Study flow chart.

**Figure 2:**
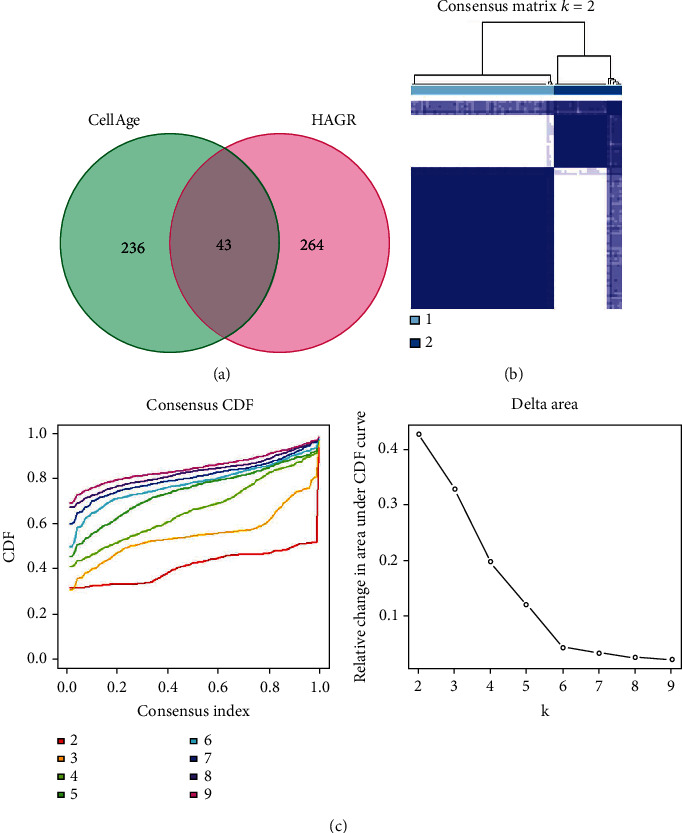
Identification of subtypes linked to aging in osteosarcoma. (a) 543 aging-related genes from CellAge and Human Ageing Genomic Resources (HAGR) database. (b) Consensus clustering solution map (*k* = 2). (c) The corresponding change in the area under the cumulative distribution function (CDF) curve for *k* = 2 to 9 is indicated by the consensus clustering-based Delta area curve.

The workflow was drawn (Figure 1(a)), and 543 ARGs were identified in total from the CellAge and HAGR database (Figure 2(a)). The ARGs clusters of individuals with osteosarcoma were determined by employing the consensus clustering method based on the aforementioned genes. The value of (clustering variable) *k* = 2 results in similarity in values in the same group, while group-to-group variation in the values exists (Figures 2(b) and 2(c)). Therefore, individuals with osteosarcoma in the TARGET cohort were categorized into Cluster 1 (57 samples) and Cluster 2 (27 samples) with distinct ARG expression patterns.

### 3.2. Functional Enrichment Analysis

**Figure 3:**
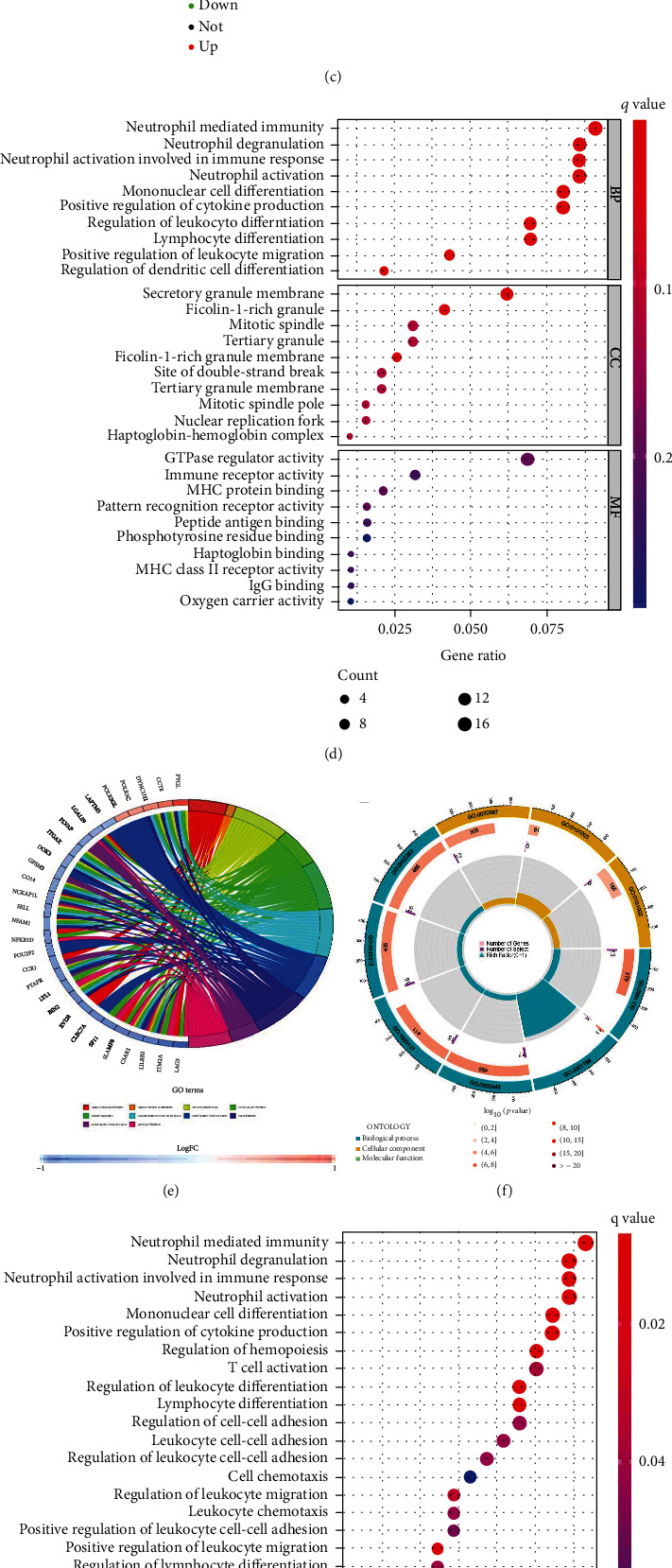
Functional enrichment analysis. (a) Kaplan-Meier curves of OS in Cluster 1 and Cluster 2 subtypes. (b) Gene Set Enrichment Analysis (GSEA) for determination of the underlying signaling pathway in Clusters 1 and 2 subtypes. (c) Volcano plot presents DEGs between Cluster 1 and Cluster 2 subtypes with threshold of |log2 FC| >0.5 and *P* < 0.05. Bubble diagram (d), chord plot (e), and circle plot (f) of Gene Ontology (GO) enrichment analysis by 278 DEGs. Bubble diagram (g), chord plot (h), and circle plot (i): Kyoto Encyclopedia of Genes and Genomes (KEGG) enrichment analysis by 278 DEGs.

The Kaplan-Meier survival curves (Figure 3(a)) indicated that the OS status of the Cluster 1 subtype was considerably poorer as compared to the Cluster 2 subtype (*P* = 0.023). The Gene Set Enrichment Analysis (GSEA) analyzed the enrichment of signaling pathways associated with the immune system in the Cluster 2 group, including IgA production using the intestinal immune pathway, cytokine-cytokine receptor interaction, and primary immunodeficiency, as well as B cell and T cell receptor signaling pathways (Figure 3(b)). The molecular mechanism between two clusters was examined using 278 DEGs which included 119 upregulated and 159 downregulated genes utilizing the Limma R package (Figure 3(c)). The GO analysis (Figures 3(d)–3(f)) and KEGG analysis (Figures 3(g)–3(i)) depicted the enrichment of DEGs in functions linked to immunity, including neutrophil-mediated immunity, neutrophil activation involved in immune response, T cell activation, B cell differentiation, and immune receptor activity. This analysis demonstrated that the Cluster 2 subtype was closely linked to the increased immune activity in the microenvironment.

### 3.3. Immune Landscape Analysis between Cluster 1 and Cluster 2 Subtype

**Figure 4:**
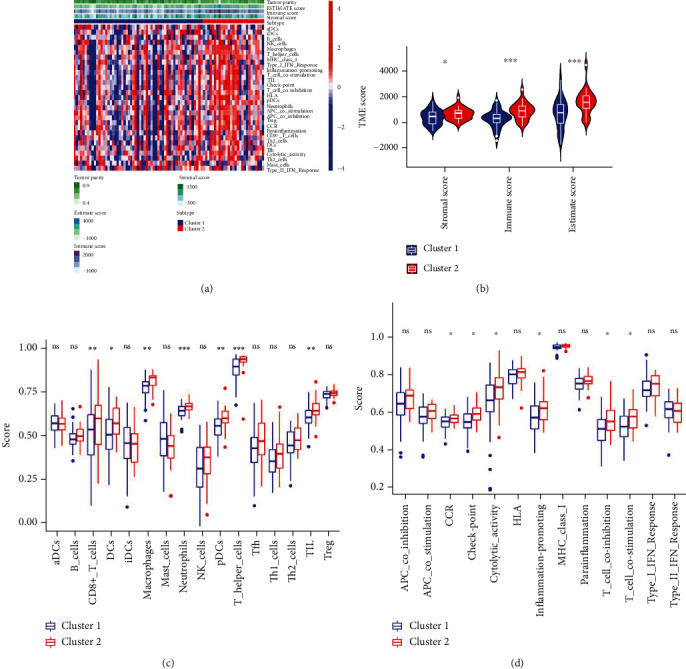
Immune landscape between Cluster 1 and Cluster 2 subtypes. (a) The heatmap shows the tumor microenvironment score, infiltration level of immune cells, and pathways related to the immune system in Clusters 1 and 2 subtypes. (b) Violin plots show the StromalScore, ImmuneScore, and ESTIMATEScore in Clusters 1 and 2 subtypes. (c) Box plots present the infiltration levels of different immune cells in Clusters 1 and 2 subtypes. (d) Box plots present the different immune-related pathways in Clusters 1 and 2 subtypes.

The above findings were taken into consideration, and the composition of the TME and immune-related function between the two subtypes was analyzed (Figure 4(a)). The ImmuneScore and StromalScore were higher in Cluster 2 compared to the Cluster 1 subtype (Figure 4(b)), indicating that Cluster 2 harbored more immune cells and stromal components. The variation in the TME of both the subtypes was analyzed by conducting a ssGSEA in each sample, which resulted in an increased enrichment of immune-related cells (including CD8+ T cells, macrophage, and helper T cells; Figure 4(c)) and immune-related functions (Figure 4(d)) in Cluster 2 subtype.

### 3.4. Construction and Validation of an ARG Signature for Osteosarcoma

**Figure 5:**
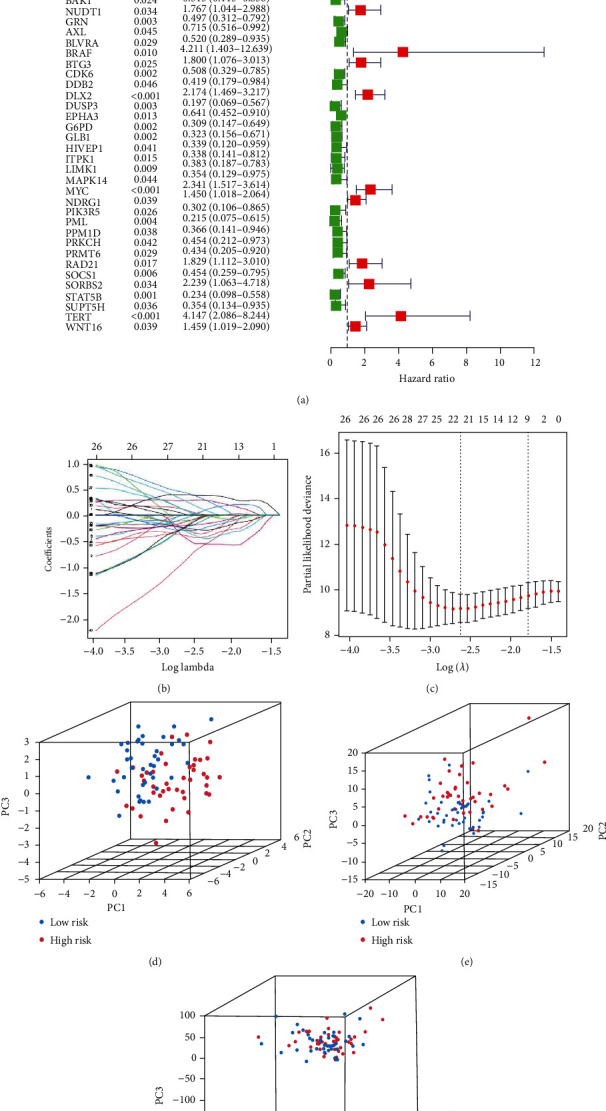
Construction of an aging-related risk model with prognostic value in osteosarcoma. (a) Forest plot utilizing the univariate Cox analysis to depict the prognosis-related aging-associated genes linked to OS. (b and c) The Least Absolute Shrinkage and Selection Operator (LASSO) regression analysis; the super parameter value was validated by means of 10-fold cross-validation. Principal component analysis (PCA) of genes in the signature (d), all aging-related genes (e), and the whole genome (f).

**Figure 6:**
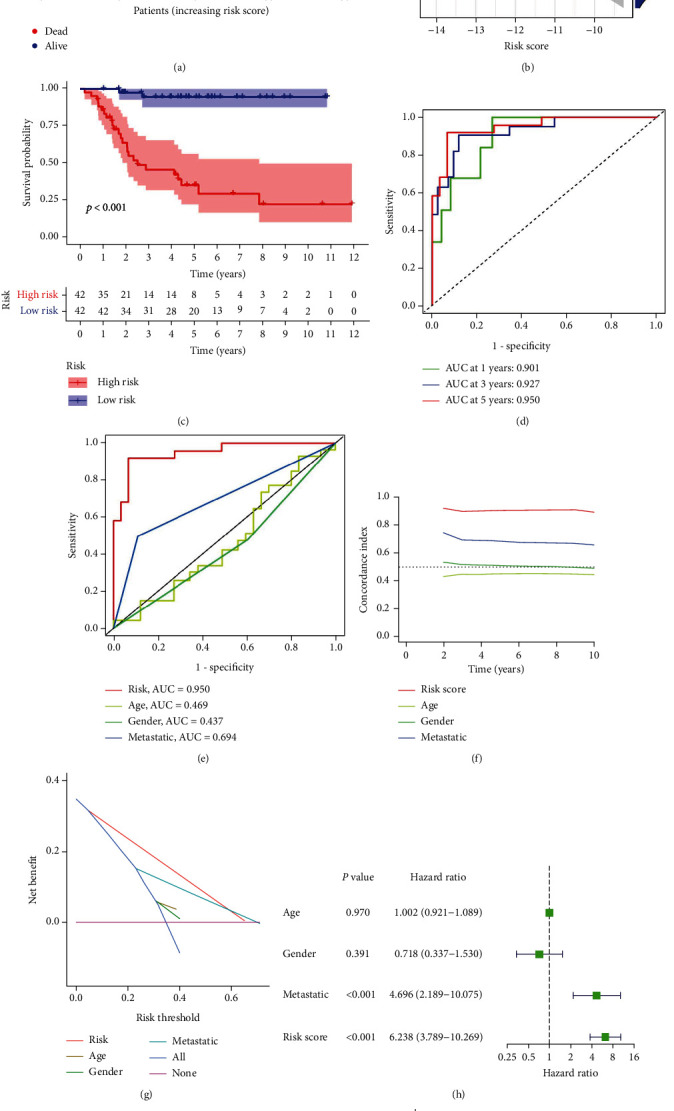
Validation of the gene signature linked to aging in the TARGET cohort. (a) Risk score, survival time, and survival status of individuals with osteosarcoma in the TARGET cohort. (b) The correlation between the risk score and survival time in the TARGET cohort. (c) Kaplan-Meier survival curve generated on the basis of an aging-related gene signature in the TARGET cohorts. (d) Risk model's ROC curve for 1-, 3-, and 5-year OS in the TARGET cohort. (e) ROC curve of the risk score, age, gender, and metastasis. (f) C-index for the risk score, age, gender, and metastasis. (g) Decision curve analysis (DCA) for the risk score, age, gender, and metastasis. Univariate (h) and multivariate (i) Cox analyses assess the risk model's independent prognostic value for individuals with osteosarcoma in the TARGET cohort using Cox analyses.

**Figure 7:**
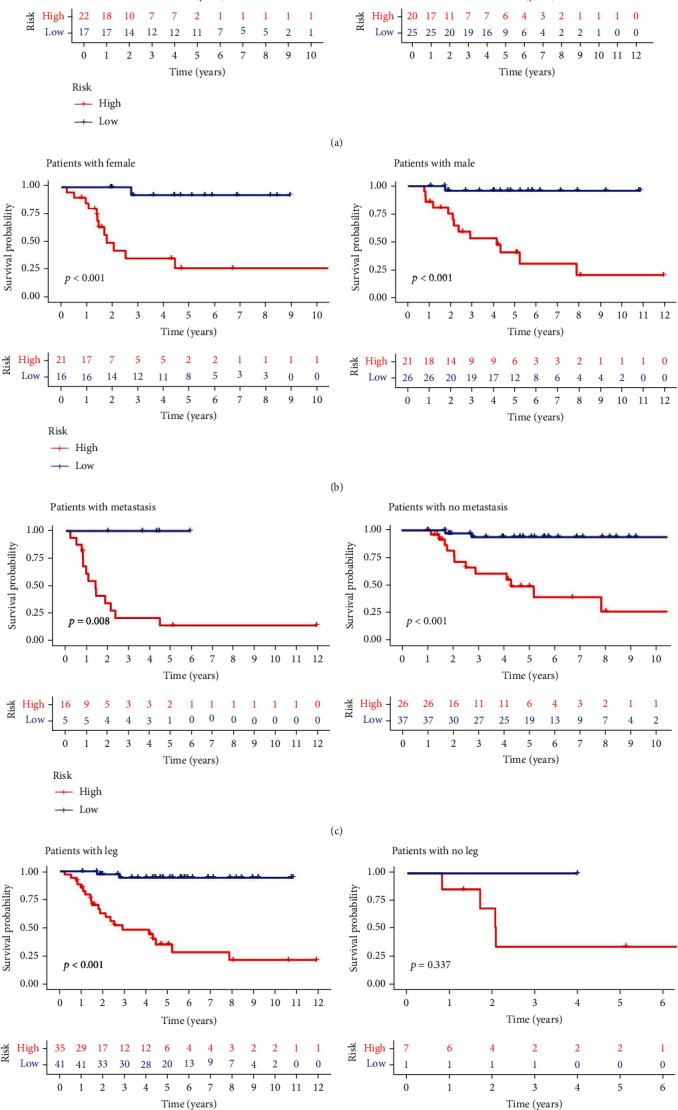
Kaplan-Meier survival curves in subgroup analyses on the basis of various clinical variables. (a) Subgroup survival analysis of risk model per age. (b) Subgroup survival analysis of risk model per gender. (c) Subgroup survival analysis of risk model as per metastatic status. (d) Subgroup survival analysis of risk model as per primary tumor site.

**Figure 8:**
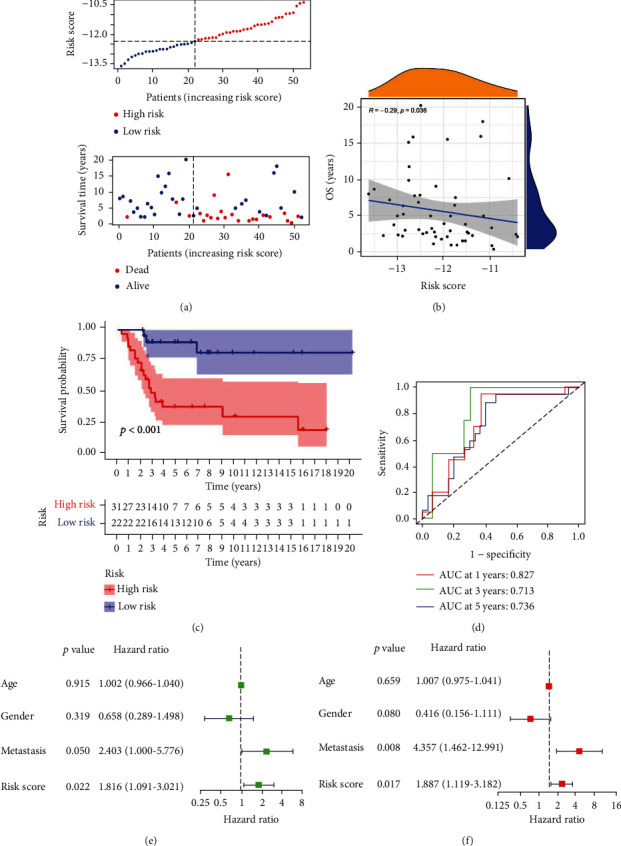
Verification of the aging-related gene signature in the GSE21257 cohort. (a) Risk score, survival time, and survival status of individuals with osteosarcoma in the GSE21257 cohort. (b) Correlation analysis between the risk score and survival time in the GSE21257 cohort. (c) Kaplan-Meier survival curve on the basis of an aging-related gene signature in the GSE21257 cohorts. (d) ROC curve of the risk model for 1-, 3-, and 5-year OS in the GSE21257 cohort. Evaluation of the independent prognostic value of the risk model using univariate (e) and multivariate (f) Cox analyses for osteosarcoma patients in the GSE21257 cohort.

The univariate Cox analysis resulted in the identification of 52 ARGs that showed a considerable link to OS for individuals with osteosarcoma (Figure 5(a)). Afterward, a total of 23 ARGs were identified as hub genes to establish the ARG signature for osteosarcoma utilizing the LASSO analysis and the multivariate Cox regression (Figures 5(b) and 5(c)). Each sample was scored using the formula, and the coefficients were shown in Table 1S. Afterward, the patients were then classified based on the median value into the two risk groups. The PCA analysis demonstrated that ARGs in the signature (Figure 5(d)) could discriminate and differentiate the two risk groups to a higher degree as compared to all ARGs (Figure 5(e)) and the whole genome (Figure 5(f)). The OS time demonstrated a negative link to the risk score (*r* = −0.58) (Figures 6(a) and 6(b)), which can be depicted using the Kaplan-Meier analysis that illustrated a link between the high-risk group and a shorter OS time compared to the low-risk group (Figure 6(c)). The risk model was further evaluated for its prediction accuracy by deriving the area under the curve (AUC). The AUCs of the 1-, 3-, and 5-years OS yielded the following respective values of 0.901, 0.927, and 0.950 (Figure 6(d)), which outperformed the AUCs obtained with clinicopathological variables (Figure 6(e)), including age, stage, and metastasis with respective values of 0.469, 0.437, and 0.694. C-index (Figure 6(f)) and DCA analysis (Figure 6(g)) also confirmed that the prediction capacity of the risk score outperformed that of age, stage, and metastasis. The risk score could also function as an independent predictor of the poor OS of patients with osteosarcoma, as demonstrated by the univariate (Figure 6(h)) and multivariate (Figure 6(i)) Cox analyses. The survival probability and the risk score relationship in age (Figure 7(a)), gender (Figure 7(b)), metastasis (Figure 7(c)), and tumor site (Figure 7(d)) subgroup were also investigated. The analysis indicated that the OS duration of the patients with the higher risk score was shorter in each subgroup, except in the no leg group, possibly due to too small sample size. Subsequently, the validation cohort was set as 53 osteosarcoma patients in the GSE21257 where the risk score was negatively linked to OS time (*r* = −0.29, Figures 8(a) and 8(b)). In this cohort, the link between the higher risk score of patients and the poor survival rate was established using the Kaplan-Meier analysis (Figure 8(c)). The respective AUCs of the 1-, 3-, and 5-years OS were 0.827, 0.713, and 0.827 (Figure 8(d)). These results were further verified by the univariate Cox analysis that linked the risk score values with the OS rates (Figure 8(e); HR = 1.816, *P* = 0.022). Furthermore, multivariate Cox analysis indicated a poor prognosis for individuals with osteosarcoma who demonstrated higher risk scores (Figure 8(f); HR = 1.887, *P* = 0.017).

### 3.5. The ARG Signature Performed Better than Others in Prognostic Prediction for Osteosarcoma

**Figure 9:**
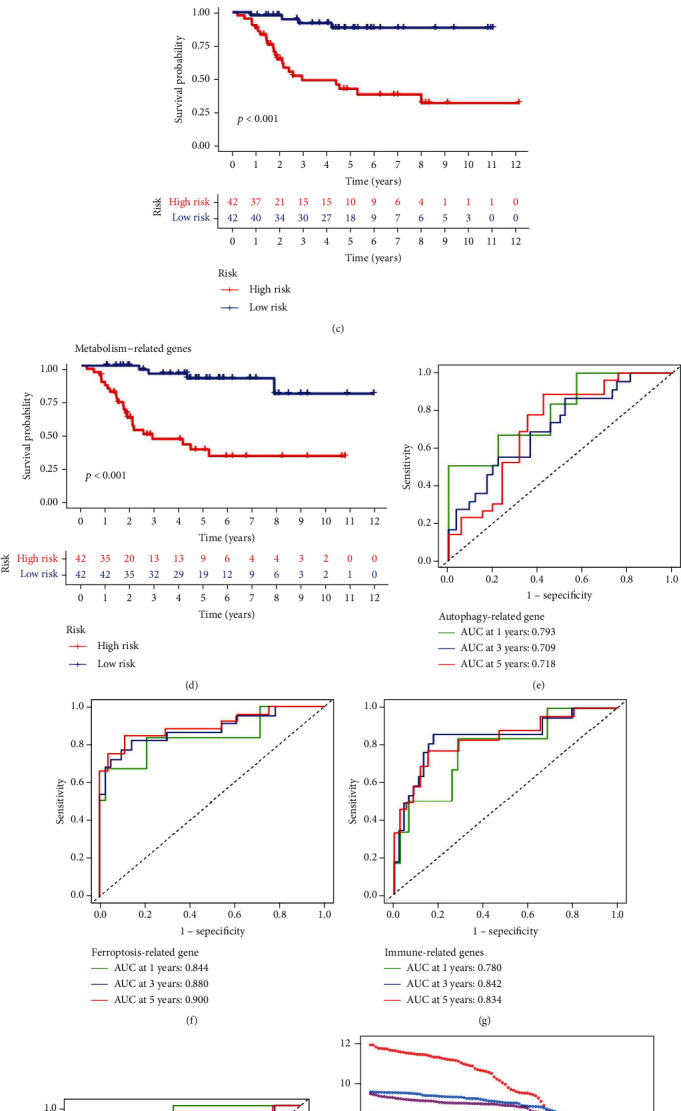
Comparison of the performance of the constructed seven-gene signature to previous signatures in the TARGET cohort. Kaplan-Meier survival analysis of autophagy-related genes signature (a), ferroptosis-related genes signature (b), immune-related genes signature (c), and metabolism-related genes signature (d). Time-dependent ROC curves of autophagy-related genes signature (e), ferroptosis-related genes signature (f), immune-related genes signature (g), and metabolism-related genes signature (h). (i) Restricted mean survival time (RMST) curve for all signatures. (j) C-index for all signatures.

The ARG prognosis model was compared with four other previously published gene signatures to examine their relative performance in prognosis prediction such as autophagy-related [20], ferroptosis-related [21], immune-related [22], and metabolism-related genes signatures [23]. Although these gene signatures were effective in creating two subgroups with considerably varied prognostic outcomes for the patients (Figures 9(a), 9(c), 9(e), and 9(g)), however, the ROC curve analysis and restricted mean survival time (RMST) values indicated the superiority of the model developed in this research. The aforementioned models depicted lower values of AUC as calculated by the former analysis for 1-, 3-, and 5-year survival compared to this model (Figures 9(b), 9(d), 9(f), and 9(h)), while this model had the highest C-index at 0.905 as calculated by RMST and obtained after comparison with the other models (Figures 9(i) and 9(j)).

### 3.6. Construction and Validation of the Nomogram Based on the Risk Model

**Figure 10:**
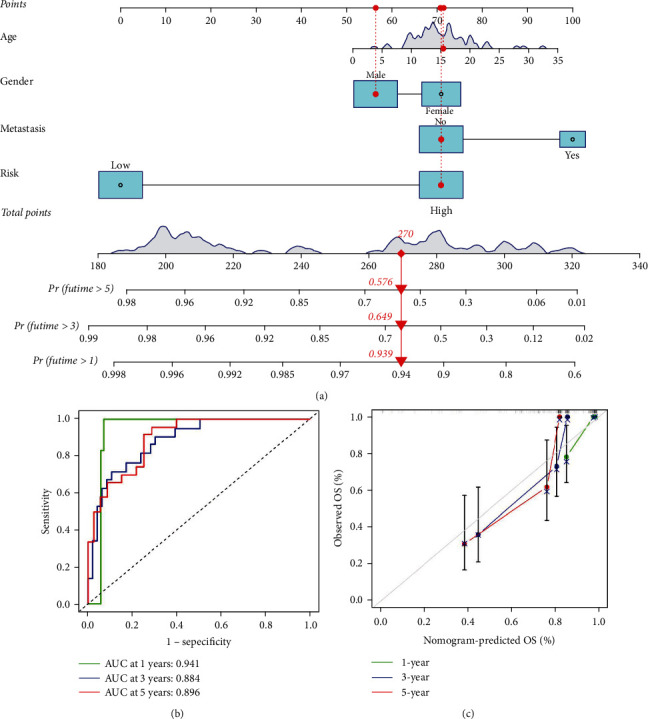
Predicting the outcome of patients with osteosarcoma using nomograms. (a) Nomogram established by the risk score, age, gender, and metastasis. (b) Nomogram's ROC curves for prediction of OS over 1, 3, and 5 years. (c) Calibration curves of the nomogram for prediction of 1-year, 3-year, and 5-year OS.

To enable the nomogram to give a very accurate prediction, the clinical factors such as sex and age of the individual as well as tumor metastasis and tumor site were integrated into the prognostic signature (Figure 10(a)). The respective AUC of the 1-, 3-, and 5-year nomograms were 0.941, 0.884, and 0.896 (Figure 10(b)). The performance of nomograms was visualized utilizing the calibration curves for 1-, 3-, and 5-year OS, where the 45° line stands for the most accurate prediction ability. The closer the calibration curves for 1-, 3-, and 5-year were to the ideal curve, the better the nomogram performed (Figure 10(c)).

### 3.7. Correlation between Risk Model and Clinicopathological Parameters, as well as Infiltrating Immunocyte Fractions

**Figure 11:**
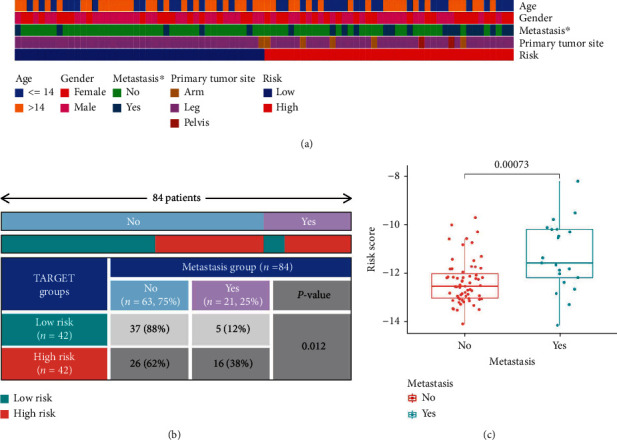
The correlation of the risk score with the clinical characteristics of osteosarcoma. (a) The heatmap of clinicopathological parameters in the high- and low-risk groups in the TARGET cohort. (b) The association between risk score and metastatic status by the chi-squared test. (c) Box plot presents the higher risk score in the metastasis patients than no metastasis patients by the Wilcoxon signed-rank test.

**Figure 12:**
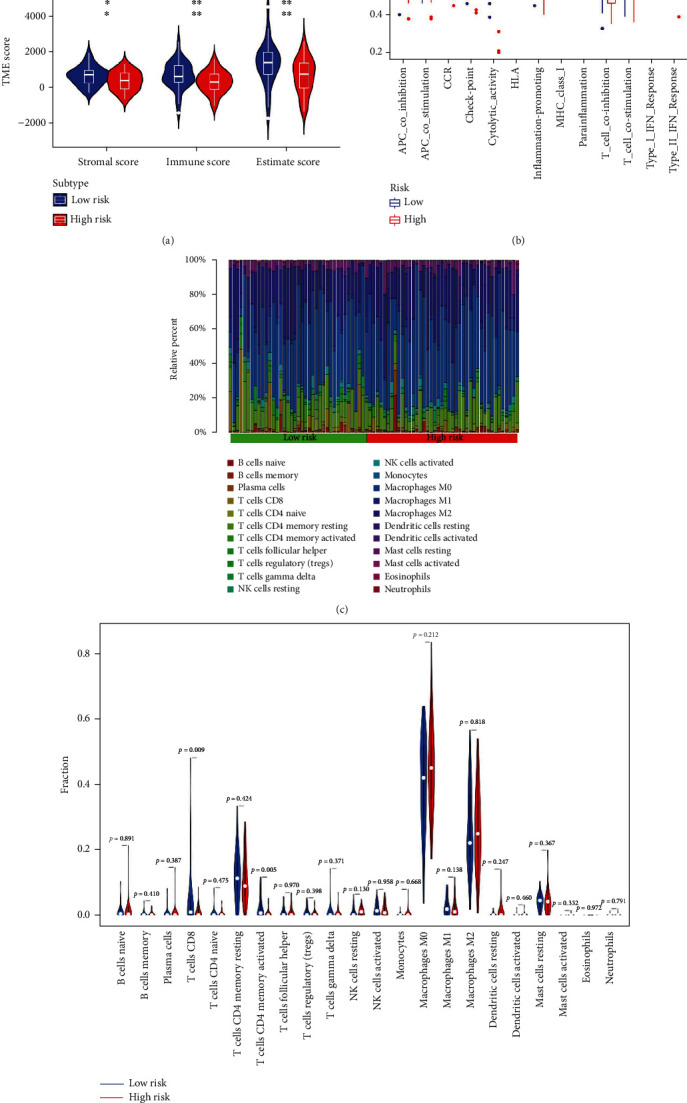
Immune landscape between the high-risk and low-risk groups. (a) Violin plots show the StromalScore, ImmuneScore, and ESTIMATEScore between the high-risk and low-risk groups. (b) Box plots present the difference in immune-related pathways between the high-risk and low-risk groups. (c) Relative proportion of infiltration levels of immune cells in the high-risk and low-risk groups. (d) Violin plot illustrates the considerable variation in immune cells between both risk groups.

The heatmap (Figure 11(a)) showed the clinicopathological parameters of patients in the two risk groups. The chi-squared test (Figure 11(b)) and Wilcoxon signed-rank test (Figure 11(c)) confirmed that the risk scores in individuals experiencing metastasis were increased as compared to those with no metastasis. The increased TME scores (including the StromalScore, ImmuneScore, and ESTIMATEScore) of the group with low-risk scores were further analyzed by TME analysis (Figure 12(a)), indicating that of the two risk groups, the one with the lower risk scores had an increased infiltration level of immune cells as compared to the group with higher risk scores. The analysis of the immune-related function through ssGSEA in the two risk groups demonstrated a higher level of enrichment of these functions in the group with low-risk scores. The level of infiltration of immune cells was analyzed through CIBERSORT where the immune cells were plotted using a bar graph to estimate their percentage in each risk group (Figure 12(c)). The abundance of activated memory CD4+ T cells and CD8+ T cells was significantly increased in the low-risk group (Figure 12(d)).

### 3.8. Immunotherapy Response Prediction

**Figure 13:**
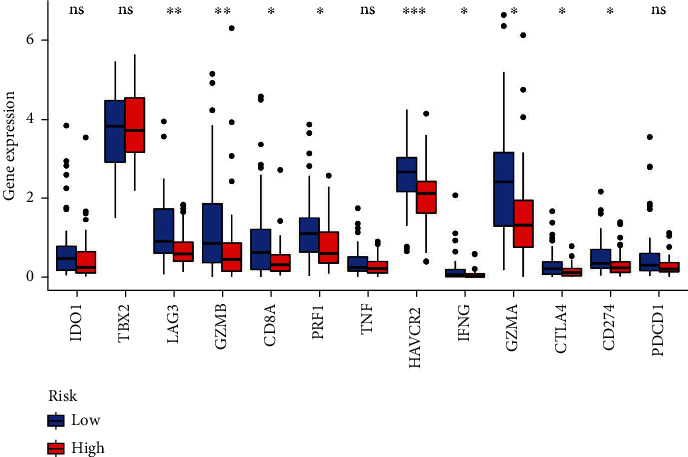
Differential expression of multiple immune checkpoint genes between the high- and low-risk groups.

The correlation between the risk model and the expression of genes related to the immune checkpoints was studied, which indicated an enhanced immune activity in the TME that led to arresting the tumor growth in the low-risk group. The enhanced immunological activity was seen due to an increase in the expression levels of genes associated with the immunological checkpoints such as *PD-L1* (*CD274*), *CTLA4*, *LAG3*, *GZMB*, *CD8A*, *PRF1*, *HAVCR2*, *IFNG*, and *GZMA* (Figure 13). These results demonstrate the increased effectiveness of immunotherapy in targeting the immune checkpoints in the group with low risk scores.

## 4. Discussion

Osteosarcoma is a common malignant tumor originating from bone tissue in children and adolescents. Osteosarcoma has a high degree of invasion and potential for distant metastases and is prone to hematogenous metastases at the early stage and after surgery, especially in the case of lung metastases [25, 26]. In recent years, despite great progress in surgery and adjuvant chemotherapy, such patients still exhibit a poor prognosis, with a high recurrence rate [27]. Therefore, identifying effective prognostic markers for risk stratification of osteosarcoma patients to adopt more aggressive interventions is expected to improve OS and might serve as potential therapeutic targets.

Aging is characterized by the accumulation of damage to macromolecules and cell architecture resulting in a progressive decrease in the function of tissue and organ due to nutrition, genetic and environmental factors, and lifestyle [28]. The accumulation of these damaged arrested cells was observed with the increase in age [29], and these senescent cells were noted to contribute to diseases that are related to aging, such as renal damage [30], alcoholic fatty liver disease [31], cerebrovascular disorders [32], diabetes [12], and Alzheimer's disease [33]. Cellular senescence is an inherent process that inhibits tumor progression, contributing to arresting the cells autonomously in the cell cycle and preventing further divisions. This process also causes the removal of these damaged cells by activating the immune system through the SASP, but if the cells evade this fate, it may lead to tumorigenesis [34]. Emerging evidence showed that several ARGs may be the cause of onset and advancement of cancers due to regulation of the process of aging and cellular senescence by these ARGs and could be used as a target for cancer therapy [35, 36]. Consequently, to analyze the exact role that aging plays in osteosarcoma, the transcriptome of the ARGs needs to be investigated thoroughly.

In the current study, using the unsupervised consensus clustering analysis, two subtypes in the TARGET cohort were determined that were related to aging, and both groups exhibited considerably varied outcomes regarding the prognosis. Furthermore, various analyses gave results that were favorable for Cluster 2 that indicated an enrichment of the signaling pathways associated with the immune response and an enhanced infiltration rate of immune cells in the TME. The GSEA was carried out for the former to determine the extent of the response by the immune system as those values could be used for observation of the progression of cancer [37]. In addition, the active immune response led to a good prognosis of the Cluster 2 subtype. The TME is an intricate network of immune cells, tumor cells, and stromal cells that contribute to tumor biology and therapeutic response. An increased infiltration level due to increased enrichment of immune effector cells was detected in Cluster 2 during the analysis of the TME, such as CD8+ T cells, macrophages, and helper T cells which can act as a protective factor against multiple cancers, such as epithelial ovarian cancer [38], head and neck squamous cell carcinoma [17, 39], and non-small-cell lung cancer [40]. These partly explain that patients belonging to the Cluster 2 subtype had a higher antitumor immune response and good prognosis.

The construction of an accurate and efficient model for cancer monitoring has become a research hotspot due to advances in RNA-sequencing and bioinformatics tools. Research has shown a considerable correlation of several ARG signatures with the prognosis of cancers such as Zhang et al. [41], Wang *et al.* [42], and others who developed gene signatures. In both cases, gene signatures that were aging-related were designed to evaluate the potential prognosis prediction efficiency of a biomarker for malignancy and study the effect of chemo- and immunotherapies. The former studied lung adenocarcinoma, while the latter studied rectal cancer. In other studies, results similar to these were detected such as in malignant melanoma [43] and lung squamous carcinoma [44]. Nevertheless, there are fewer studies about the function of ARGs as a prognosis-determining factor in osteosarcoma. In this research, an ARG signature was demonstrated to better predict the prognosis of osteosarcoma than conventional clinicopathological characteristics utilizing LASSO regression and multivariate Cox regression analysis. The risk scores and metastasis could predict the patients' prognosis independently as depicted by the univariate and multivariate Cox regression analyses, which was consistent with the finding in the validation cohort. Moreover, compared to previously reported signatures for osteosarcoma prognosis [20–23], the time-dependent ROC curve analysis and C-index revealed a better ability of this ARG model to predict the prognosis of patients with osteosarcoma. Subsequently, using risk scores and clinical characteristics, a nomogram was developed that depicted more convenient usage in clinical settings. The nomogram exhibited better performance than the single ARG risk model in predicting short-term OS. Based on the above finding, the ARG risk model was a promising novel prognostic marker and could improve individualized treatment strategy.

Emerging immunotherapy, including anti-CTLA4 [45], anti-LAG3 [46], anti-PD-1, and anti-PD-L1 antibodies [47], has been proven to be efficacious and increased the survival rate of patients with several advanced cancers, including metastatic osteosarcoma patients [48]. However, considering the heterogeneity and complexity of osteosarcoma, only a few patients had a favorable response to immunotherapy [49]. The components and activity of TME are critical determinants of the response to immunotherapy [50]. As compared with other cancers, osteosarcoma has low immune infiltration in TME, which may be one reason for unsatisfactory immunotherapy results [49, 51]. In this research, the group with the lower risk scores had increased StromalScore, ImmuneScore, and immune functions, indicating that the risk group with low scores demonstrated increased immune infiltration level and immunogenicity in comparison with the high-risk group, which probably contributed to the better survival outcomes. The CIBERSORT analysis showed that CD8+ T cells and activated memory CD4+ T cells were more infiltrated in the low-scoring risk group. CD8+ T cells are essential in the antitumor activity and are a favorite prognosis marker for osteosarcoma patients [52]. The CD8+ T cells can be differentiated into cytotoxic T lymphocytes (CTLs) by CD4+ T cells through multiple mechanisms, as well as maintaining and enhancing the antitumor response of CTLs [53]. Intriguingly, CD4+ T cells have been identified as having direct antitumor cytolytic function [54]. The efficiency of blockade therapy based on immune checkpoints is primarily dependent on the expression of genes associated with immune checkpoints and T cell-dependent immune response [55]. Unsurprisingly, in the low-risk group, the immune checkpoint genes were expressed more, particularly PD-L1, CTLA4, and LAG3, showing that the low-risk patients could be more benefited from the immune checkpoint blockade therapy. Therefore, this study indicates that the ARG signature may be useful in filtering patients who can benefit from immunotherapy.

This study had some limitations. Both the TARGET-OS cohort and the GSE21257 cohort have relatively small sample sizes, and the finding based on bioinformatics analysis was insufficient for clinical practice. Therefore, the results need to be verified by utilizing large study samples and *in vitro* or *in vivo* experimental verification. Furthermore, the specific functions of ARGs in the signature in osteosarcoma remain ambiguous and need more study.

## 5. Conclusion

In conclusion, this study identified two aging-related subtypes and an ARG prognostic signature that depicted robust performance in the prognosis prediction of osteosarcoma patients, which might help in guiding clinical management. Furthermore, ARG prognostic signature showed the different immune landscapes for osteosarcoma patients, which guide the personalized application of immunotherapy. However, before applying aging-related subtypes and prognostic signatures, these findings need to be verified by more clinical samples.

## Figures and Tables

**Table 1:** Clinical characteristics of the individuals with osteosarcoma in this research.

Covariates	Type	Target		GSE21257	
		Number	Percent	Number	Percent
Age	≤14	39	46.43%	15	28.30%
	>14	45	53.57%	38	71.70%
Gender	Female	37	44.05%	19	35.85%
	Male	47	55.95%	34	64.15%
Race	White	51	60.71%	—	—
	Asian	6	7.14%	—	—
	Black or African American	7	8.33%	—	—
Primary tumor site	Leg	76	90.48%	44	83.02%
	Arm	6	7.14%	8	15.09%
	Pelvis	2	2.38%	—	—
Metastatic status	Yes	21	25.00%	14	26.42%
	No	63	75.00%	39	73.58%
Survival status	Dead	27	32.14%	23	43.40%
	Alive	57	67.86%	30	56.60%

## Data Availability

The TARGET datasets and Gene Expression Omnibus database provided the supporting data for the conclusions drawn in this study and can be accessed at their websites: (https://ocg.cancer.gov/programs/target) and (https://www.ncbi.nlm.nih.gov/geo), respectively.

## Supplementary Materials

Supplementary Materials Table 1S: the coefficients of aging-related genes in the signature.
