# Supplementary material for: Identification and Development of an Age-Related Classification and Signature to Predict Prognosis and Immune Landscape in Osteosarcoma
Source: J Oncol. 2022 Oct 12;2022:5040458. doi: 10.1155/2022/5040458 (PMC9581613; doi:10.1155/2022/5040458)
Supplement: Supplementary Materials — Table 1S: the coefficients of aging-related genes in the signature. [file 5040458.f1.docx]

| Gene | Coef |
| --- | --- |
| PTPN1 | -0.08646 |
| INSR | 0.377868 |
| CEBPA | -0.07365 |
| MXI1 | 0.380322 |
| GTF2H2 | -0.3422 |
| ARHGAP1 | -0.38452 |
| ERCC4 | -0.54638 |
| PPARG | -0.27081 |
| EPS8 | -0.1236 |
| BAK1 | -0.08182 |
| GRN | -0.2969 |
| BTG3 | 0.00947 |
| CDK6 | -0.18693 |
| DDB2 | -0.12139 |
| DLX2 | 0.047718 |
| G6PD | -0.0062 |
| HIVEP1 | -0.17748 |
| MYC | 0.291187 |
| PPM1D | -0.69514 |
| RAD21 | 0.028407 |
| STAT5B | -0.43045 |
| TERT | 0.027046 |
| WNT16 | 0.185946 |
